# The performance of a new type accelerator uRT‐linac 506c evaluated by a quality assurance automation system

**DOI:** 10.1002/acm2.14226

**Published:** 2023-11-27

**Authors:** WenZhao Sun, ZhongHua Shi, Xin Yang, SiJuan Huang, Can Liao, Wei Zhang, YongBao Li, XiaoYan Huang

**Affiliations:** ^1^ State Key Laboratory of Oncology in South China Collaborative Innovation Center for Cancer Medicine Sun Yat‐sen University Cancer Center Guangzhou China; ^2^ Guangdong Esophageal Cancer Institute Guangzhou China; ^3^ Radiotherapy and Imaging R&D department Shanghai United Imaging Healthcare Co., Ltd. Shanghai China

**Keywords:** EPID, machine performance, multi‐leaf collimator, quality assurance automation

## Abstract

**Purpose:**

The purpose of this study was to evaluate the performance of our quality assurance (QA) automation system and to evaluate the machine performance of a new type linear accelerator uRT‐linac 506c within 6 months using this system.

**Methods:**

This QA automation system consists of a hollow cylindrical phantom with 18 steel balls in the phantom surface and an analysis software to process electronic portal imaging device (EPID) measurement image data and report the results. The performance of the QA automation system was evaluated by the tests of repeatability, archivable precision, detectability of introduced errors, and the impact of set‐up errors on QA results. The performance of this linac was evaluated by 31 items using this QA system over 6 months.

**Results:**

This QA system was able to automatically deliver QA plan, EPID image acquisition, and automatic analysis. All images acquiring and analysis took approximately 4.6 min per energy. The preset error of 0.1 mm in multi‐leaf collimator (MLC) leaf were detected as 0.12 ± 0.01 mm for Bank A and 0.10 ± 0.01 mm in Bank B. The 2 mm setup error was detected as −1.95 ± 0.01 mm, −2.02 ± 0.01 mm, 2.01 ± 0.01 mm for X, Y, Z directions, respectively. And data from the tests of repeatability and detectability of introduced errors showed the standard deviation were all within 0.1 mm and 0.1°. and data of the machine performance were all within the tolerance specified by AAPM TG‐142.

**Conclusions:**

The QA automation system has high precision and good performance, and it can improve the QA efficiency. The performance of the new accelerator has also performed very well during the testing period.

## 1 INTRODUCTION

Modern radiotherapy techniques involve multiple simultaneous motions along different axes and these increasingly complex systems increase quality assurance (QA) requirements. American Association of Physicists in Medicine (AAPM) Task Group 142 report (TG 142)^1^ and TG 198 report (an extension of TG 142)^2^ highlight the importance of routine QA of medical linear accelerators and describe a series of important tests to be performed at regular intervals. The QA program goal is to assure that the machine characteristics do not deviate significantly from their baseline values acquired at the time of machine acceptance and commissioning because the deviation from baseline values could result in suboptimal treatment of patients. Nowadays many new advanced technologies^3^, ^4^, ^5^, ^6^ and various imaging devices^7^, ^8^, ^9^, ^10^ are integrated as common parts of the medical linear accelerator. Therefore, the QA test methods become more and more complex and needed to spend a lot of time executing. This is a very heavy workload for physicists, especially considering that some require manual operation. This leads to an urgent need for QA automation system that all tests should be streamlined, simple, rapid, and reproducible.

Automatic machine QA may be the direction and trend in the future.^11^ At present, the research of automating QA in clinic is increasing daily.^12^, ^13^, ^14^, ^15^, ^16^, ^17^, ^18^, ^19^, ^20^ In 2015 Eckhause^21^ developed an automating QA program to perform machine mechanical tests using both electronic portal imaging device (EPID) images and log files, and the whole delivery and analysis took approximately 30 min to complete. Clivio^22^ firstly evaluated the machine performance check (MPC) on TrueBeam linear accelerator in 2015. The tests focused on machine geometry and beam performance were executed automatically with a single plan. The whole time for acquiring all required images and data analyzing was about 6 min. In 2016 Jenkins^23^ developed a novel QA automation system to perform mechanical and geometric tests using a radio‐luminescent phosphor coated phantom and optical images. The system was able to automatically collect, analyze and report the results for all tests. And the total time was less than 10 min for whole procedure. After that, a large number of literatures on evaluation of MPC were published.^24^, ^25^, ^26^, ^27^, ^28^, ^29^ The publication of these articles showed the feasibility of automating QA for medical linear accelerator and provided a large number of available test methods. The system was composed of a standard phantom and an automatic processing software. Although these systems had their own advantages, they were all designed for a certain type of machines and could not be used directly on the other machines. Moreover, the extension of the test encompassing more QA items also is needed. In addition, allowing the development of other QA automation systems with competitive algorithms is also conducive to the research of automating QA.

The purpose of this study was to design a new QA automation system to perform routine machine QA on a new medical linear accelerator uRT‐linac 506c (Shanghai United Imaging Healthcare Co., Ltd, UIH) using a cylindrical phantom built in‐house and acquired EPID images. The QA items included MLC/EPID/Jaw/couch positioning precision, collimator/gantry/couch rotation isocenter, angle accuracy, the beam consistency and laser localization accuracy. We also assessed this QA automation system with repeatability, precision, detectability of introduced errors, the impact of set‐up errors on QA results, and the machine performance of this new medical linear accelerator at semi‐annual period based on our QA automation system.

## 2 MATERIAL AND METHODS

The QA automation system we proposed consisted of a cylindrical phantom and an automatic analysis software. It emphasized the mechanical requirements of AAPM TG142 and TG198. The software was developed with MATLAB (The Mathworks, Inc., Natick, MA) to automate the acquisition and analysis of EPID images.

All QA item testing plans are integrated into one QA plan for automatic and continuous execution of QA programs. The machine performance of this new linear accelerator over a semi‐annual period was evaluated using this QA automation system, as well as the repeatability, precision, the detectability of introduced errors, and the impact of set‐up errors on QA results of this QA automation system were also evaluated.

### 2.1 Linear accelerator and EPID system

The uRT‐linac 506c is a C‐arm linear accelerator with a 16‐slice helical Computed Tomography (CT) imager coaxially attached behind. The diameter of enclosed CT bore is 70 cm and distance between CT origin and RT isocenter is 210 cm. The linear accelerator has two energy modes: 6 MV flattened beam (flattening filter, FF) with a maximum dose rate of 600 MU/min and 6 MV unflattened beam (flattening filter free, FFF) with a maximum dose rate of 1400 MU/min. The collimator contains dual‐layer jaws at top and 60 pairs of MLCs at the bottom with 40 narrow central leaves projecting to 0.5 cm and 20 outer leaves projecting to a width of 1.0 cm at the isocenter plane. The MV Cone Beam Computer Tomography (CBCT) system employing a 1.5 MeV photo beam.

The uRT‐linac 506c includes an integrated amorphous silicon electronic portal imaging device (aSi‐EPID) with an active area of 41 cm × 41 cm perpendicular to the treatment beam. The model Varex AP16 EPID has a resolution of 1024 × 1024 with an approximately 0.4 × 0.4 mm pixel size. All images in this study were acquired at a source to image distance (SID) of 145 cm. The image has a resolution of approximately 0.28 × 0.28 mm at the isocenter plane.

### 2.2 BB phantom

**FIGURE 1:**
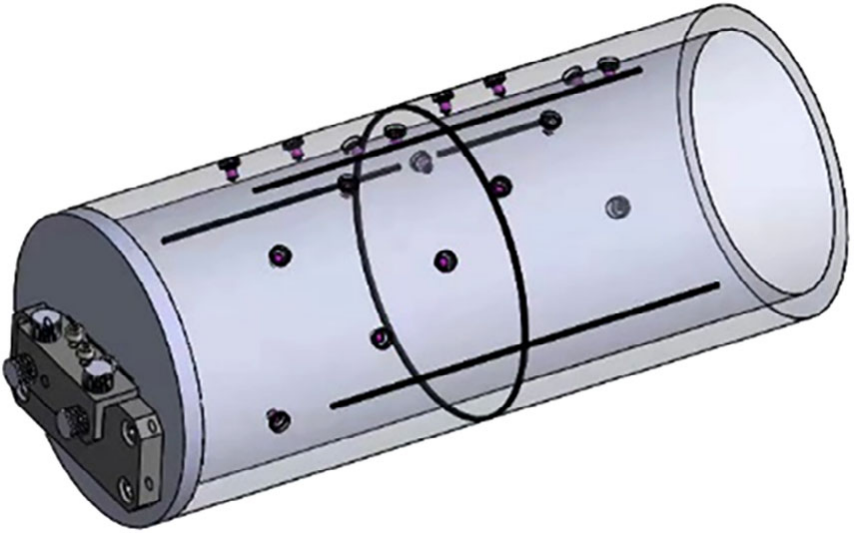
The schematic view of the BB phantom structure.

The BB phantom is built in‐house. It is a hollow cylinder with a diameter of 13 cm and a length of 30.9 cm, and is constructed of polymethyl methacrylate (PMMA) with a total 18 steel balls with 4‐mm diameter embedded into the surface. It is mounted to the couch top using a dedicated holder when performing QA. The spatial coordinate position of the steel ball relative to the phantom center is established. This standard phantom with established structures can standardize the tests and enable automated analysis. Figure 1 shows a schematic view of the BB phantom structure.

### 2.3 Software and analysis algorithms

The automating QA software can be used for evaluating the performance of MLC, EPID, jaw, treatment couch, gantry, laser, isocenter and beam consistency. In order to monitor the change of system state, it is necessary to acquire the baseline images after the commissioning and acceptance of the linear accelerator. The baseline images include correction field image, MLC field images, jaw field images. The process of acquiring the baseline images is similar to the performing automating machine QA. All images are automatically imported into in the software for analysis.

#### 2.3.1 Self‐calibration

**FIGURE 2:**
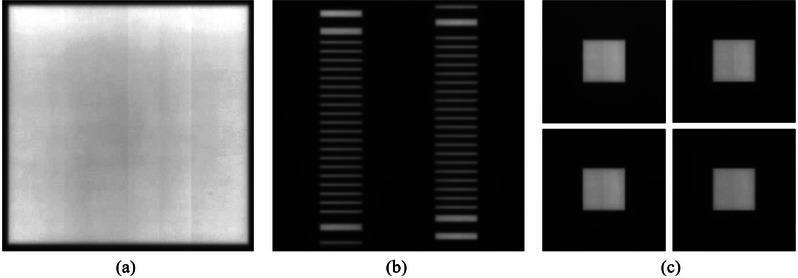
The calibration beam image. (a) open field beam image; (b) comb‐shaped beam image; (c) the square beam images with four principal collimator angles.

In the process of performing machine QA, the mechanical deformation from EPID and non‐uniformity response of EPID detectors have a great impact on the analysis results. Therefore, in order to reduce these influences on the analysis results, the calibration beams were added into the QA plans for the purpose of correcting the EPID image. The calibration beams are shown in Figure 2, including an open field image (a), comb‐shaped beam image (b) and square field rotation images (c).

When acquiring the calibration beams, the BB phantom will be automatically moved out of the radiation field through the couch movement controlled by the QA plans. The open field image is used for bad pixel correction and response correction of EPID detectors. Abnormal points will be identified and replaced by the mean values of surrounding points. Correction of response consistency is based on the symmetry principle of the radiation field. The comb‐shaped field image is used to determine the central pixel row where the center of each MLC leaf is located, the rotation angle correction and horizontal correction of EPID plate, and the scaling coefficient of EPID image. The square field images are acquired at 0°, 90°, 180° and 270° of the collimator, respectively. The position of the rotation center on the EPID is determined by fitting the field center under different collimator angles. All these calibration beam images needed to be acquired prior to the acquisition of QA beam images so that calculated various correction coefficients could be stored and directly used to correct the images acquired in the subsequent QA procedure.

#### 2.3.2 MLC

**FIGURE 3:**
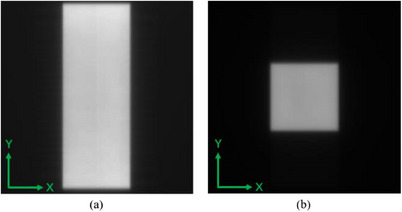
The beam shape patterns. (a) MLC beam image; (b) jaw beam image.

The MLC QA test employes a specific field size beam image as shown in Figure 3(a) to evaluate the change of positioning accuracy of central 46 pairs of leaves. The longitudinal position (Y direction) of MLC leaf is determined by the parameters calculated in Section 2.3.1. The transversal position (X direction) of MLC leaf end is determined by the position where the image gray gradient of each leaf changes the most. In order to improve the positioning accuracy, every original pixel grid is equally subdivided to 10 parts which the sub‐pixel grid is about 0.038 mm by cubic spline interpolation or Gaussian fitting. The offset of each MLC leaf is calculated by comparing with the baseline. The maximum leaf offset and average leaf offset of each leaf bank, MLC field center offset and MLC field size deviation are all statistically calculated to monitor the state change of MLC.

#### 2.3.3 JAW

The jaw position check is performed using a specific size field shown in Figure 3(b). The analysis method of X and Y jaw is similar to that of MLC. The same interpolation method is used to improve the jaw position accuracy. The jaw absolute position is calculated based on the seven lines near the center. The calculated offset of each jaw and jaw field center offset were used to monitor the state change of jaw.

#### 2.3.4 EPID

**FIGURE 4:**
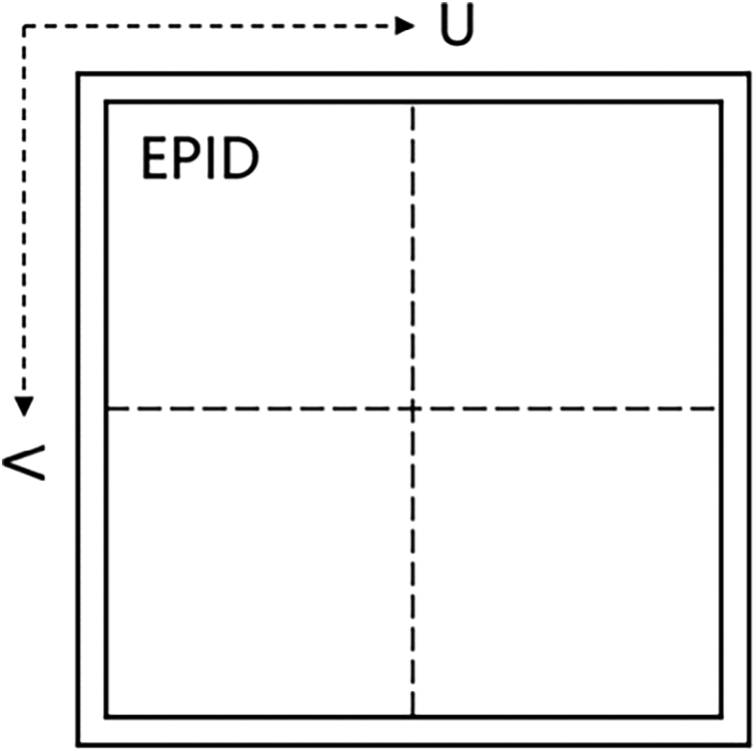
The EPID coordinate system (U/V). The arrow indicates the positive direction.

In this part, the images acquired in the self‐calibration stage were used for analysis. The rotation center was calculated in the EPID coordinate system shown in Figure 4. The source‐to‐image distance (SID) was also calculated using the scaling coefficient of EPID image multiplied by the nominal SID of 145 cm. And the corresponding offsets to the baseline was also calculated, respectively.

#### 2.3.5 Collimator

**FIGURE 5:**
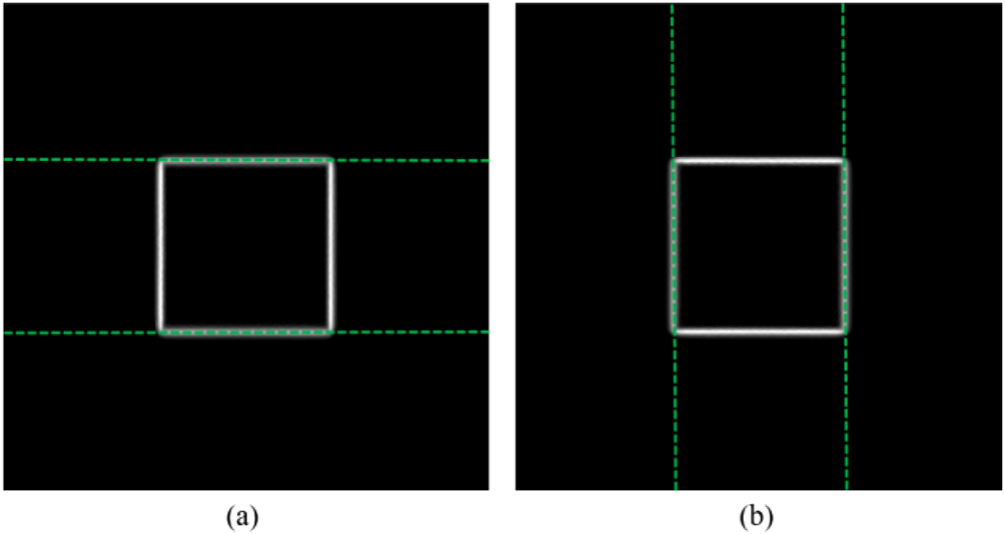
The gradient image of 0° collimator with fitting lines of beam edges (a) and the gradient image of 90° collimator with fitting lines of beam edges (b).

The offset of angle rotation and zero‐degree angle are two important QA indicators. In this process, the images of the rotation field acquired in the self‐calibration process are used for analysis. As shown in Figure 5, the field edge is fitted by gradient transformation of beam image and the tilt angle of fitting line is calculated. The offset of zero‐degree is calculated by averaging the two tilt angles, respectively, from two parallel fitting lines on the image of 0° collimator relative to the baseline. The rotation offset is obtained by calculating the change of fitting line from same edge of the image from 0° to 90° of collimator angle relative to the nominal value. It's also the average of the calculated angular offset of the two edges.

#### 2.3.6 Beam consistency

Before the irradiation, the five dark field images needed to be acquired to remove the influence of EPID residual signal. Afterwards, 10 same raw images were acquired with a field size of 25 cm × 25 cm and 1 MU, which the beam stability had been verified through 2D array detector measurements. After stacking these 10 images, the bad pixel correction, detector gain correction (remove the inconsistency of response of each pixel), median filtering and mean filtering were performed on the superimposed images. In order to remove the influence of the mechanical repeatability of EPID itself, the field center was aligned with the EPID image center and image gray values were adjusted with the scaling coefficient because the gray value is inversely proportional to the square of source to image distance (SID).

**FIGURE 6:**
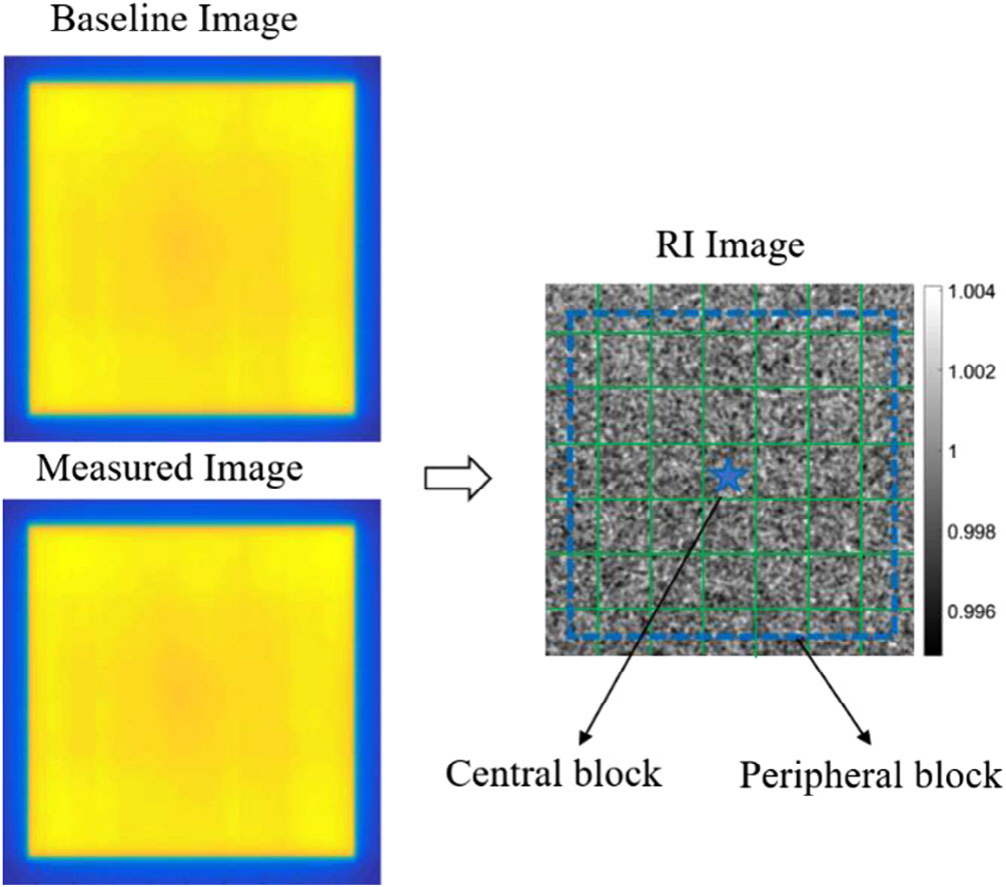
Baseline image, measured image and ratio image (RI) in beam QA.

The ratio image (RI) was the ratio of pixel values between the measured image and the baseline image at the same coordinates as shown in Figure 6. And 20 cm × 20 cm of RI central area (the 80% of the filed size) were used for analysis. This area was divided into 7 × 7 blocks and the mean pixel values of each block which contains about 10000 pixels were calculated for analysis. The mean values in the central block were used for calculating the dose output change (OC). The differences of mean pixel values between every peripheral block and the central block reflected the changes of energy spectrum. The maximum difference was used to characterize the change of beam uniformity (UC) which might be caused by changes in energy or magnetic field deflection, etc. The threshold of the beam uniformity is set at ±1%. The field center offset was calculated by comparing the rotation center obtained in self‐calibration process with the baseline. All the formulas are expressed as follow.

(1) $$OC=\left(\sum_{\left(x,y\in CROI\right)}RI\left(x,y\right)/\;N_{CROI}-1\right)*100\%$$

(2) $$\left\{\begin{array}{c} UC\;=\max\left(\left|\mathrm{ECDif}f_{1}\right|,\left|\mathrm{ECDif}f_{2}\right|,\text{…}\text{…}\left|\mathrm{ECDif}f_{i}\right|\right)\;\\ \;\mathrm{ECDif}f_{i}=\;\sum_{x,y\in EROI_{i}}\frac{RI\left(x,y\right)}{N_{EROI_{i}}}-\sum_{x,y\in CROI}\frac{RI\left(x,y\right)}{N_{CROI}} \end{array}\right.$$

where $N_{CROI}\;$represents the number of pixels in the central block. $N_{EROI_{i}}$ represents the number of pixels in each peripheral block. ECDiff_i_ represents the difference of gray level change between peripheral block and central block.

#### 2.3.7 Isocenter

**FIGURE 7:**
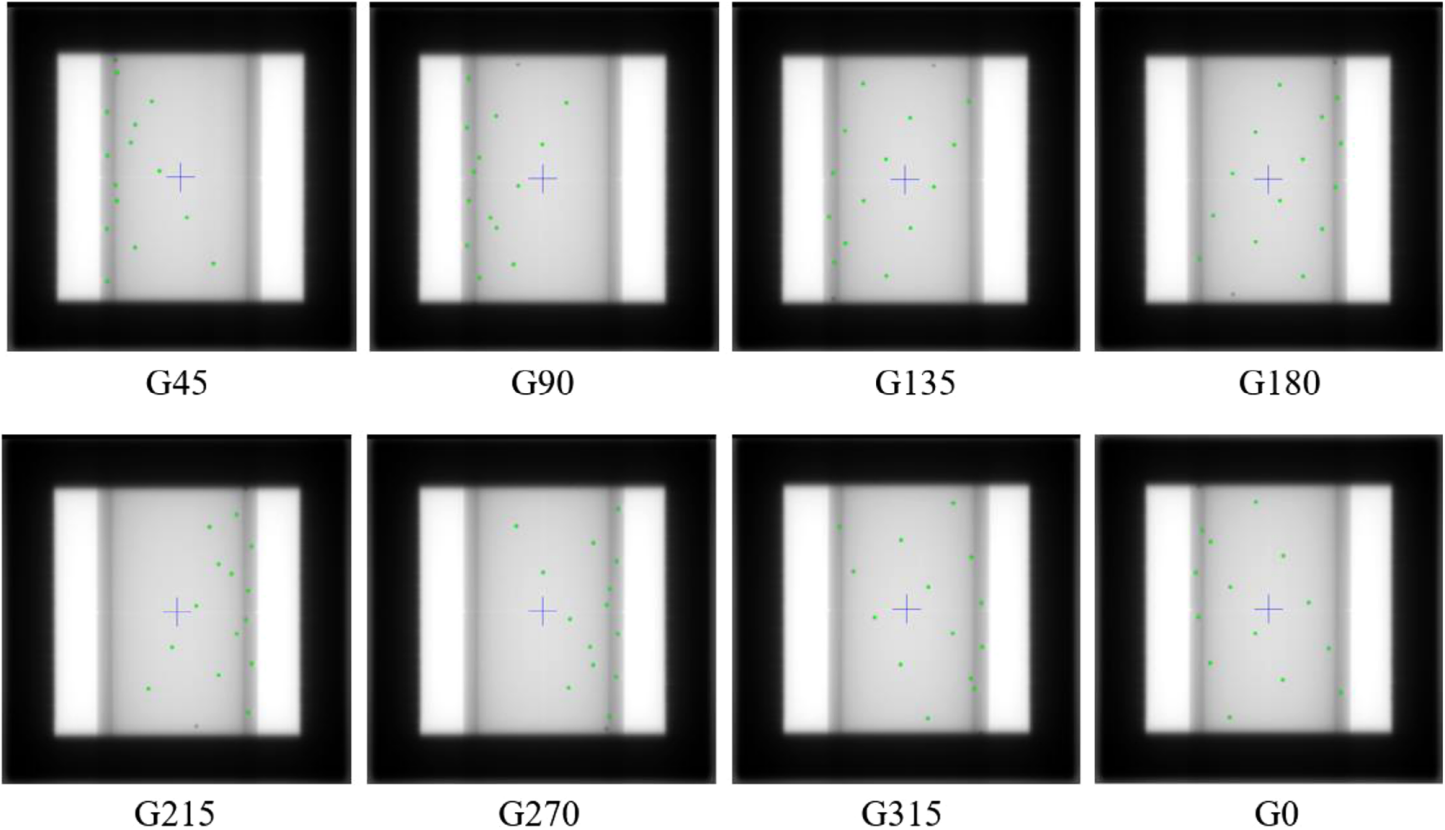
Square field and BB phantom images acquired at different gantry angles.

When performing the isocenter QA, the BB phantom was moved to the preset couch position according to the QA plan. The eight images with a 20 cm × 20 cm square field formed by jaw as shown in Figure 7 were acquired at the gantry angle of 45°, 90°, 135°, 180°, 215°, 270°, 315°, 0°, respectively.

In the acquired images, the projection position of the steel ball on the phantom and the field center were marked with green point and blue crossline as shown in Figure 7, respectively. The projection coordinate matrix P_mat_ was established by using the actual spatial coordinate position (*x_i_,y_i_,z_i_*) and the projection pixel position (*u_i_,v_i_*) of the steel ball on the BB phantom. The equation was shown as follows:

(3) $$\left[\begin{array}{c} \lambda u_{i}\\ \lambda v_{i}\\ \lambda \end{array}\right]\;\;=\;\;Pmat*\;\left[\begin{array}{c} x_{i}\\ \begin{array}{c} y_{i}\\ z_{i}\\ 1 \end{array} \end{array}\right]=\left[\begin{array}{ccc} p_{11} & p_{12} & \begin{array}{cc} p_{13} & u_{0} \end{array}\\ p_{21} & p_{22} & \begin{array}{cc} p_{23} & v_{0} \end{array}\\ p_{31} & p_{32} & \begin{array}{cc} p_{33} & 1 \end{array} \end{array}\right]\;\left[\begin{array}{c} x_{i}\\ \begin{array}{c} y_{i}\\ z_{i}\\ 1 \end{array} \end{array}\right]$$

The P_mat_ matrix was calculated by using the spatial relationship established by the coordinate positions of multiple steel balls, and the equation was solved by the least square method in the calculation process. Each gantry angle corresponded to a projection matrix, and then the radiation beam axis in space was reconstructed by using the projection pixel coordinates (*u_i_,v_i_*) of the center of the radiation field. The points on the radiation beam axis could be expressed as:

(4) $$\left[\begin{array}{c} x\\ y\\ z \end{array}\right]{\left[\begin{array}{ccc} p_{11} & p_{12} & p_{13}\\ p_{21} & p_{22} & p_{23}\\ p_{31} & p_{32} & p_{33} \end{array}\right]}^{-1}\left[\begin{array}{c} \lambda u_{1}-u_{0}\\ \lambda v_{1}-v_{0}\\ \lambda \;-\;1 \end{array}\right]$$

**FIGURE 8:**
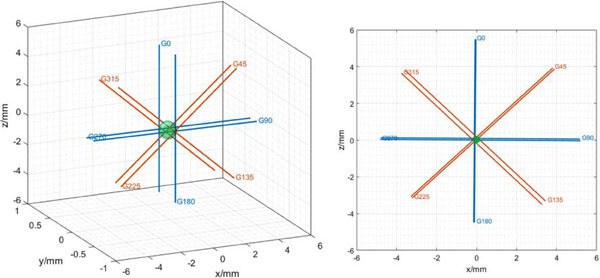
Radiation beam axis at different gantry angles (left) and its projection in XOZ plane (right).

By selecting different scale coefficients λ the coordinates of the spatial points on the radiation beam axis could be determined. Therefore, the spatial trajectory of the radiation beam axis rotating with the gantry was shown in Figure 8. The calculated diameter of the smallest ball containing all radiation beam axes represented the accuracy of the radiation isocenter. In addition, when the positioning laser was aligned with the phantom marker (at this time, the coordinate system with the phantom geometric center as the origin is aligned with the spatial coordinate), the position of the steel ball center in spatial coordinate system was used to reflect the deviation of laser from the isocenter.

#### 2.3.8 Treatment couch

**FIGURE 9:**
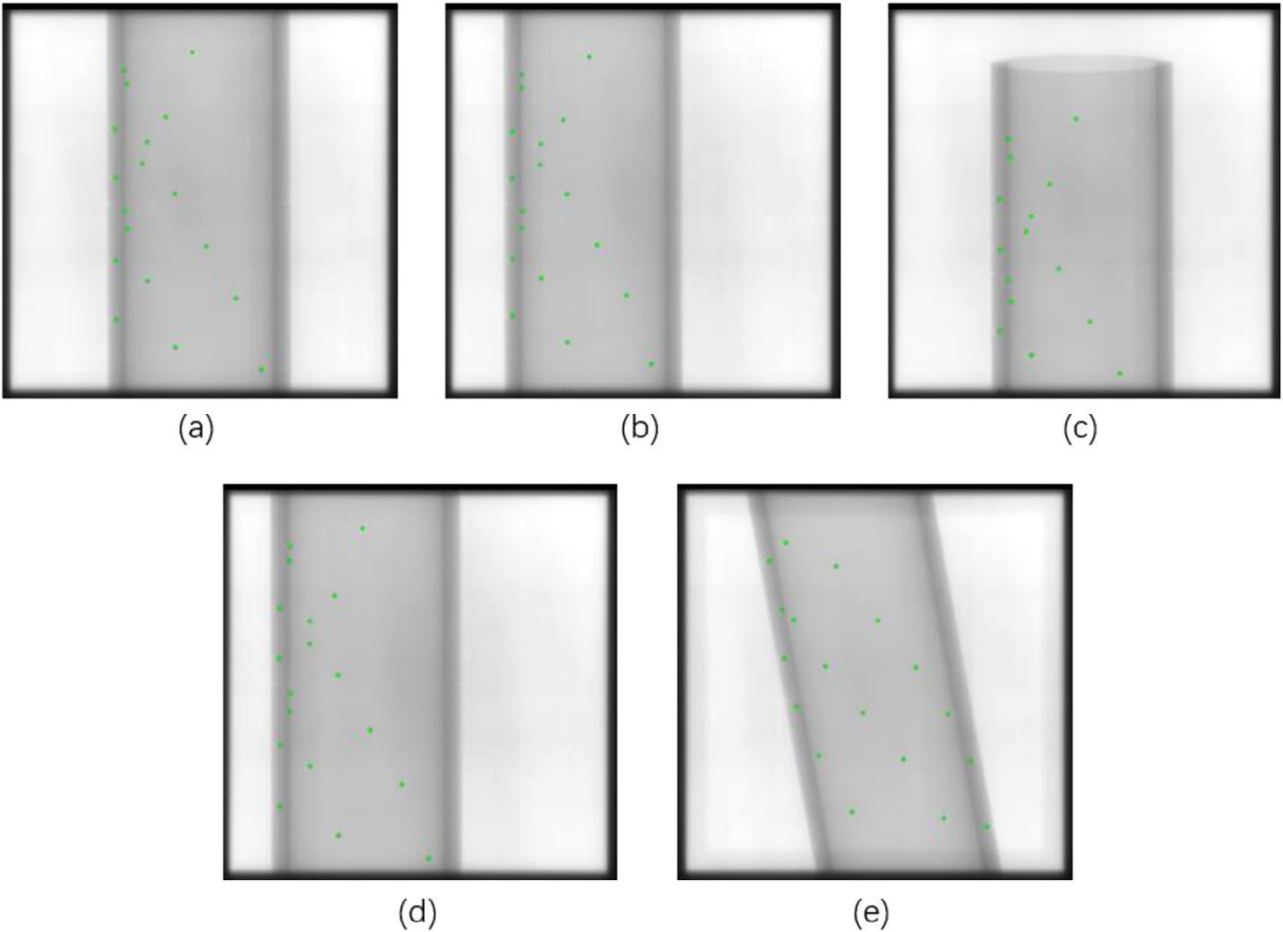
The images of BB phantom with different couch positions. (a) Image at the initial position; (b) Image after couch movement −5 cm along the X‐axis; (c) Image after couch movement −5 cm along the Y‐axis; (d) Image after couch movement along −5 cm the Z‐axis; (e) Image after couch rotation 5°.

During the QA of the treatment couch, the image was acquired at the initial position of the phantom setup with the gantry of 45° as the reference image. And then the phantom was moved an expected distance of −5 cm along each couch axis and 10° rotation and the all images as shown in Figure 9 were acquired, respectively. In order to improve the accuracy of measurement, the relationship between the change of steel ball position in space coordinate system and the change of steel ball position in projection coordinate system could be used to calculate the actual movement of each axis of the treatment couch. The change in the spatial position of the steel ball caused by the movement of the couch could be expressed as:

(5) $$\left[\begin{array}{c} x_{i}\\ y_{i}\\ z_{i} \end{array}\right]R_{z}R_{y}R_{x}\left[\begin{array}{c} x_{0}\\ y_{0}\\ z_{0} \end{array}\right]\left[\begin{array}{c} T_{x}\\ T_{y}\\ T_{z} \end{array}\right]$$

where (*x*_0,_ *y*_0,_ *z*_0_) is the initial spatial coordinates of the steel ball (the center of the phantom is the coordinate origin), *R_x_*_,_ *R_y_*, *R_z_* are the rotation matrixes of the treatment couch around each axis, *T_x_*_,_ *T_y_*, *T_z_* are the translation displacements of the couch along each axis, (*x*_i,_ *y*_i,_ *z*_i_) is the spatial coordinates of the steel ball after the movement of the couch, *R_x_*_,_ *R_y_*, *R_z_* can be expressed as:

(6) $$\left\{\begin{array}{c} \;R_{x}=\;\left[\begin{array}{cc} 1 & \begin{array}{cc} 0 & \;0 \end{array}\\ \begin{array}{c} 0\\ 0 \end{array} & \begin{array}{c} \begin{array}{cc} \cos\left(roll\right) & \sin\left(roll\right) \end{array}\\ \begin{array}{cc} -\sin\left(roll\right) & \cos\left(roll\right) \end{array} \end{array} \end{array}\right]\\ \;R_{y}=\;\left[\begin{array}{cc} \cos\left(pitch\right) & \begin{array}{cc} 0 & -\sin\left(pitch\right) \end{array}\\ \begin{array}{c} \;0\\ \sin\left(pitch\right) \end{array} & \begin{array}{c} \begin{array}{cc} 1 & \;0 \end{array}\\ \begin{array}{cc} 0 & \cos\left(pitch\right) \end{array} \end{array} \end{array}\right]\\ \;R_{z}=\;\left[\begin{array}{cc} \cos\left(yaw\right) & \begin{array}{cc} \sin\left(yaw\right) & \;0 \end{array}\\ \begin{array}{c} -\sin\left(yaw\right)\\ 0 \end{array} & \begin{array}{c} \;\begin{array}{cc} \cos\left(yaw\right) & 0 \end{array}\\ \;\begin{array}{cc} 0 & \;1 \end{array} \end{array} \end{array}\right] \end{array}\right.$$

The change of the projection position of the steel ball caused by the movement of the couch could be expressed as:

(7) $$\left[\begin{array}{c} \lambda u_{i}\\ \lambda v_{i}\\ \lambda \end{array}\right]\;\;=\;\;Pmat_{0}*\left[\begin{array}{c} x_{i}\\ \begin{array}{c} y_{i}\\ z_{i}\\ 1 \end{array} \end{array}\right]$$

where (*u*_i,_ *v*_i_) is the projection coordinate of a steel ball after the movement of the couch. Multiple steel balls could establish multiple equations and could be solved with the least square method. When the couch moved along an axis, the movement amount of the couch could be calculated by the equations established by Formulas (5)–(7).

#### 2.3.9 Gantry

**FIGURE 10:**
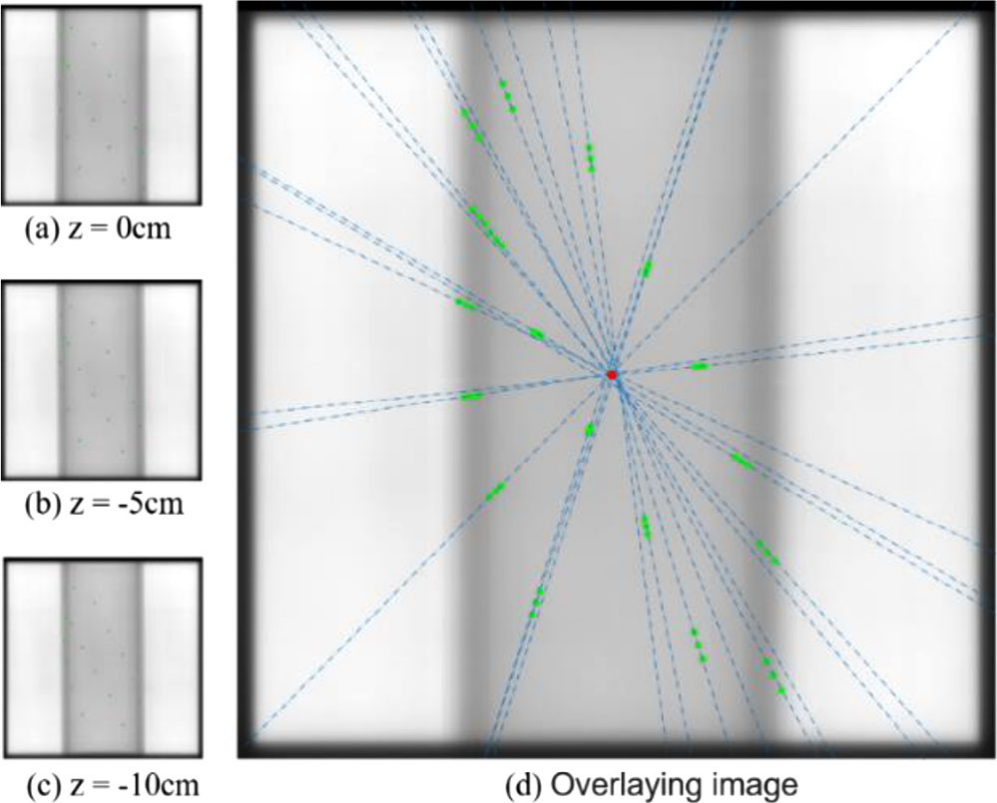
Projection image and overlaying images of BB phantom at different heights of treatment couch.

During the QA of the gantry, the BB phantom was moved to the initial position. The images at gantry angle of 0° were acquired at the z‐axis positions of 0 cm, −5 cm and −10 cm, respectively, by controlling the vertical movement of the couch. After superimposing images, the position of intersection point (Point P, as shown in the position of red mark in Figure 10) of all straight lines linked by the center of same steel ball at different couch positions was determined, this position related to the actual gantry angle. The projection matrix of BB phantom at the original position was calculated through Equation (3), and then the position of projection pixel *P*_0_ of isocenter on the imaging board was obtained by using the isocenter spatial position and projection matrix calculated in Section 2.3.7. The angle G_angle_ of the gantry could be expressed as:

(8) $$G_{angle}=arctan\left(PP_{0}/SID\right)$$

where *PP*_0_ is the distance between pixel P and pixel P0. The offset of the gantry 0° could be evaluated in the above way.

**FIGURE 11:**
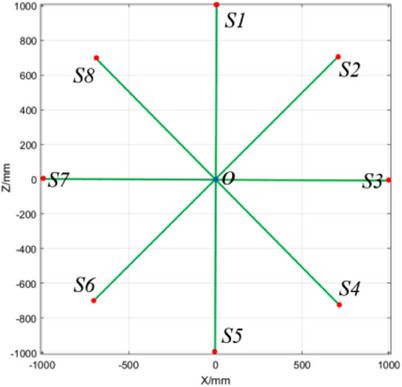
Change of focal spot position of radiation beams during gantry rotation.

In addition, using the data in Section 2.3.7 and Formula (4), when λ was taken by 0, all straight lines converged to a point, which was the focal spot position of the radiation beam. Under different gantry angles, after the spatial position of the focal spot of the radiation beam was determined, the rotation angle of the gantry could be calculated through the isocenter position calculated in Section 2.3.7, as ∠S_1_OS_2_ shown in Figure 11. The rotation accuracy of the gantry can be obtained by comparing it with the preset angle.

### 2.4 The performance tests of the QA automation system

The QA automation system was repeatedly run seven times on this new medical linear accelerator uRT‐linac 506c for repeatability testing. Considering the accuracy of the MLC leaf position can reach 0.1 mm, a system error of −0.1 mm was added to the position of all leaves in Bank A and 0.1 mm was added to the position of all leaves in Bank B in the QA testing plan for the MLC item test. The new QA test plan was run three times and compared with the baseline to test and verify the achievable precision of the QA automation system. We also modified the QA test plan to simulate actual machine errors and the detailed preset error values were listed in Table 2. This new QA plan was run repeatedly and compared with the baseline to evaluated whether the preset error could be detected by the QA automation system. The effect of phantom set‐up error was tested by modifying the couch position to move the phantom −2 mm for all direction from original position.

## 3 RESULTS

The results obtained from our automatic QA system on UIH 506C at semi‐annual period and the ground truth data were listed in Table 1. All the obtained data have small variability and are within the tolerance specified by AAPM TG‐142. All EPID images were acquired at source to image distance (SID) of 145 cm and have approximately 0.4 mm of the pixel size. When calculated at isocenter and interpolated, the accuracy of the data could reach sub millimeter. Therefore, the data uncertainty is on the order of 0.1 mm which were proven by the precision test and the repeatability test.

**TABLE 1:** The results obtained from our automatic QA system on UIH 506C at semi‐annual period.

Parameters	GT						
MLC	Mean ± SD	1st	2nd	3rd	4th	5th	6th
Mean Offset A [mm]	−0.2 ± 0.1	−0.02 ± 0.061	−0.03 ± 0.059	−0.03 ± 0.06	−0.02 ± 0.073	−0.08 ± 0.076	−0.06 ± 0.078
Mean Offset B [mm]	−0.1 ± 0.1	0.09 ± 0.066	0.04 ± 0.065	0.07 ± 0.063	0.08 ± 0.066	0.22 ± 0.073	0.16 ± 0.075
Max Offset A [mm]	<0.5 ± 0.5	−0.15	−0.15	−0.16	−0.18	−0.22	−0.26
Max Offset B [mm]	<0.5 ± 0.5	0.22	0.16	0.23	0.2	0.41	0.31
Center shift AB [mm]	0.1 ± 0.1	0.05	0.02	0.03	0.04	0.09	0.06
Field size [mm]	−0.3 ± 0.1	0.01	−0.03	0	0	0.2	0.12
EPID							
Offset X [mm]	0.06	0.01	0	−0.19	−0.15	0.04	−0.16
Offset Y [mm]	0.17	0.12	−0.25	0.01	0.05	0.03	−0.17
SID [mm]	0.2	0.02	0.08	0.06	0.05	−0.08	0.22
COLLIMATOR							
Offset of 0°[°]	0.00 ± 0.03	−0.03	−0.02	−0.01	0.02	−0.06	−0.03
Rotation [°]	0.00 ± 0.01	0.01	0.02	0.02	0.06	0.04	0.03
JAW							
Offset X1 [mm]	−0.5 ± 0.1	0	0.04	0.06	−0.04	−0.01	−0.04
Offset X2 [mm]	−0.5 ± 0.1	0.01	−0.01	−0.02	−0.08	0.03	0.01
Offset Y1 [mm]	−0.5 ± 0.1	0.05	0.11	−0.04	−0.03	0.07	0.05
Offset Y2 [mm]	−0.5 ± 0.1	0.13	0.2	0.08	0	0.29	0.1
Center shift X [mm]	0.0 ± 0.1	0.04	0.04	0.05	0.01	0.04	0.03
Center shift Y [mm]	0.0 ± 0.1	−0.12	−0.08	−0.15	−0.17	−0.07	−0.12
BEAM							
Output change [%]	−0.3 ± 0.1	0.01	−0.7	0.27	−0.52	−0.69	−0.29
Consistency change [%]	0.2 ± 0.1	0.14	0.08	0.32	0.14	0.34	0.28
Center shift [mm]	0.2 ± 0.1	0.28	0.28	0.34	0.3	0.3	0.31
COUCH							
lateral [mm]	−0.1 ± 0.1	0.16	−0.01	−0.42	−0.31	−0.64	−0.3
Longitudinal [mm]	−0.1 ± 0.1	−0.18	−0.15	−0.08	−0.17	−0.3	−0.28
Vertical [mm]	−0.1 ± 0.1	0.38	0.26	−0.01	0.09	−0.15	0.04
Rotation [°]	0.0 ± 0.1	0.06	0.04	0.04	0.05	0.07	0.05
Center shift [mm]	0.5 ± 0.1	0.06	0.04	0.17	0.21	0.03	0.12
ISOCENTER							
Isocenter size [mm]	0.35 ± 0.01	0.21	0.24	0.18	0.22	0.21	0.2
Laser shift X [mm]	0.2 ± 0.1	0.68	0.27	−0.48	0.15	0.44	0.86
Laser shift Y [mm]	−0.5 ± 0.1	−0.25	−0.2	−0.8	−0.87	1.05	0.63
Laser shift Z [mm]	0.2 ± 0.1	−0.84	−1.23	−0.72	−1.24	−0.18	−0.74
GANTRY							
Offset of 0° [°]	−0.17 ± 0.03	0.04	0.05	0.09	0.15	0.07	0.1
Rotation [°]	0.00 ± 0.01	0.01	0.09	0.11	0.06	0.01	−0.02

Abbreviations: GT, ground truth; SD, Standard Deviation.

In addition, we also evaluated the short‐term repeatability, precision, detectability of introduced errors, and the impact of set‐up errors on QA results of this QA automation system. The detailed data were shown in Table 2. The data of the short‐term repeatability test showed the data variation is within 0.1 mm and 0.1°. The precision test showed the 0.1 mm errors in MLC leaves could be detected. The data of the detectability test of introduced errors showed that the preset errors could also be accurately detected. And the impact of set‐up error on test showed that it had no effect on the analysis results when the phantom moved to position A from position C (moving −2 mm in all three directions relative to original positioning center), and the laser position deviation can be detected accurately.

**TABLE 2:** The results obtained from the tests of the repeatability, precision, detectability of introduced errors and the impact of set‐up errors on QA results of this QA automation system.

Parameter		Repeatability test	Impact of set‐up errors test	Detectability of introduced errors test		Precision test	
		Calculated	Difference		Calculated		Calculated
MLC	Thresh	Mean(M‐BL)	Mean(A‐C)	Preset	Mean ± SD	Preset	Mean(M‐BL)
Mean Offset A [mm]	±0.5	0.00 ± 0.01	0.00 ± 0.01	−0.5	−0.50 ± 0.01	−0.1	−0.12 ± 0.01
Mean Offset B [mm]	±0.5	0.01 ± 0.01	0.01 ± 0.01	0.5	0.51 ± 0.01	0.1	0.10 ± 0.01
Max Offset A [mm]	±1.0	−0.14 ± 0.02	0.01 ± 0.02	−0.5	−0.64 ± 0.02		
Max Offset B [mm]	±1.0	0.13 ± 0.02	0.04 ± 0.02	0.5	0.63 ± 0.02		
Center shift AB [mm]	±0.5	0.00 ± 0.00	0.00 ± 0.00				0.00 ± 0.00
Field size [mm]	±0.5	0.01 ± 0.02	0.02 ± 0.02	1.0	1.01 ± 0.02		0.22 ± 0.01
EPID							
Offset X [mm]	±1	−0.02 ± 0.00	0.03 ± 0.01				
Offset Y [mm]	±1	0.02 ± 0.01	−0.00 ± 0.01				
SID [mm]	±1	−0.05 ± 0.01	−0.07 ± 0.01				
COLLIMATOR							
Offset of 0°[°]	±0.5	−0.02 ± 0.02	0.02 ± 0.02				
Rotation [°]	±0.5	0.02 ± 0.02	0.01 ± 0.02				
JAW							
Offset X1 [mm]	±1	−0.01 ± 0.01	−0.07 ± 0.01	−0.5	−0.51 ± 0.01		
Offset X2 [mm]	±1	0.02 ± 0.01	0.01 ± 0.01	0.5	0.52 ± 0.01		
Offset Y1 [mm]	±1	−0.01 ± 0.01	−0.02 ± 0.01	−0.5	0.51 ± 0.01		
Offset Y2 [mm]	±1	−0.02 ± 0.03	0.03 ± 0.01	0.5	0.48 ± 0.03		
Center shift X [mm]	±0.5	0.01 ± 0.01	−0.02 ± 0.01				
Center shift Y [mm]	±0.5	−0.01 ± 0.01	−0.00 ± 0.01				
BEAM							
Output change [%]	±2	−0.34 ± 0.14	0.03 ± 0.02				
Consistency change [%]	±1	0.21 ± 0.11	0.02 ± 0.01				
Center shift [mm]	0.5	0.01 ± 0.01	0.00 ± 0.01				
COUCH							
lateral [mm]	±1	−0.05 ± 0.01	0.05 ± 0.01	1	0.95 ± 0.01		
Longitudinal [mm]	±1	0.02 ± 0.01	0.11 ± 0.01	−1	−1.02 ± 0.01		
Vertical [mm]	±1	0.00 ± 0.03	−0.01 ± 0.01	1	1.00 ± 0.03		
Rotation [°]	±0.5	−0.02 ± 0.01	−0.04 ± 0.01	0.5	0.48 ± 0.01		
Center shift [mm]	0.75	−0.01 ± 0.01	−0.02 ± 0.01				
ISOCENTER							
Isocenter size [mm]	0.5	−0.06 ± 0.10	−0.01 ± 0.02				
Laser shift X [mm]	±2	−0.01 ± 0.01	−1.95 ± 0.01				
Laser shift Y [mm]	±2	0.00 ± 0.01	−2.02 ± 0.01				
Laser shift Z [mm]	±2	0.04 ± 0.02	−2.01 ± 0.02				
GANTRY							
Offset of 0° [°]	±0.5	0.03 ± 0.01	−0.00 ± 0.01	−0.5	−0.47 ± 0.01		
Rotation [°]	±0.5	−0.06 ± 0.01	0.05 ± 0.01				

*Note*: Thresh: Threshold; SD: Standard Deviation; M‐BL: Measured data minus Baseline; A‐C: Measured data difference between position A and position B.

The time required for the acquisition of all the 40 required images and analysis was approximately 4.6 ± 0.1 min per energy. The whole process of phantom setup, test plans delivery, EPID images acquiring and processing and result presentation was within 7 min.

## 4 DISCUSSION

QA automation system is indeed a highly effective QA tool, which enable automatically QA plans delivery, images acquisition and data analysis. The total of 40 images acquisition takes about 4.6 min per energy. Comparing with 5.6 min needed for MPC to acquire 39 images per energy,^22^ our method improves the efficiency by nearly 20%. This may be due to the lower MUs and the optimized trajectory of the radiation field. In addition, our system analyzed more QA items, 31 items compared with 23 items of MPC. This may be due to the strategy of compressing the number of images and extracting more information from the same image as much as possible.

The QA automation system is mainly based on the images acquired by EPID for analysis. The inconsistent response of each pixel of EPID and the offset, tilt, rotation of EPID plate^30^ could directly affect the quality of analysis results. Different from the fixed position of the treatment couch adopted by MPC, our QA automation system uses the correction factor generated by the self‐calibration method to correct the acquired images during data processing, so as to improve the accuracy of the results and ensure the repeatability of the measurement.

In the stage of phantom setup, there will always be a certain setup error when we align the phantom according to the location laser. Considering the variability of each phantom setup errors and the potential impact on the result, we have also designed the novel algorithm to avoid the impact of the phantom setup error on the result, which was also proved by our verification experiments with the data listed in Table 2. The results are only reflected in the position offset of the laser localization. The preset positioning error of 2 mm in each direction can be accurately identified by the system.

The purpose of developing this automatic QA system is to evaluate the overall performance of this new linear accelerator efficiently and accurately. According to our QA data obtained at semi‐annual period, all indicators are within the tolerance recommended in AAPM TG‐142 report.^1^ Some of the results such as MLC offset and jaw offset are far less than the tolerance, which may be due to the good performance of the machine itself and the high‐resolution advantage of EPID image itself, and higher accuracy of 0.1 mm order can be obtained after interpolation.

For the performance of MLC in the analyzed period, our data showed the mean offsets were not larger than 0.4 mm. It may be related to the MLC performance and the MLC leaf identification algorithm. Eckhause^21^ found the MLC positioning accuracy on two TrueBeam accelerator to be 0.000 ± 0.039 mm with EPID measurements for static measurement based on their own QA automation system. In contrast, Clivio's research^22^ showed positioning accuracy of MLC was within 0.5 mm using MPC for evaluating the performance of TrueBeam accelerator. Another interesting finding is that, the offsets of all leaves in bank A are negative direction and the offsets of all leaves in bank B are positive direction. This result is similar with the published literature.^15^ The systematic deviation indicates that no stochastic error takes place and may potentially be caused by imperfections in the alignment or calibration of the MLC leaf tracking system. Since the average offset of leaf in bank B is greater than that of leaf in bank A, the center of the radiation field formed by MLC is also shift toward the direction of bank B. In addition, the offset of MLC leaf position towards the outside also leads to the increase of the actual field size. However, slight leaf offsets could not have a significant clinical impact on the delivery of beam.^15^

There are only slight deviations for checks of jaw, collimator, EPID and beam consistency in the analyzed period. Similar to the results reported in other literatures,^21^, ^22^, ^23^, ^26^ no significant systematic deviation is observed for QA of jaw, collimator and EPID, which indicates that the linear performance of positioning calibration and angle calibration is very good. One of the possible reasons is that our algorithm adopts the method of gradient transformation of EPID image to avoid the impact of inconsistent pixel response on the results. The inconsistent response of pixels has an important impact on the beam consistency analysis. Therefore, we reduce this impact by performing relative response calibration. Of course, we regularly monitor the beam output and calibrate it based on the measurement of ionization chamber, so as to ensure that the fluctuation of output is in a small range. At present, there is no widely accepted quantitative evaluation index for the change of the 2D shape of the beam relative to the baseline data. Clinically, we usually judge by observing the coincidence of the 1D profile shape of the two beams. In this system, we try to establish a novel quantitative evaluation method. Through this index, we observed that the beam of this new linear accelerator has good stability.

The QA of gantry angle, beam isocenter and treatment couch is performed under different gantry angles. According to the relevant research report,^30^ under different gantry angles, EPID has different displacements due to gravity. Our algorithm uses the projection coordinates of the spatial structure of phantom as the reference, so as to avoid the influence of EPID displacement on the results, such as in the evaluation of field rotation isocenter and gantry rotation angle accuracy. In addition, the method of gantry angle 45 degrees can easily realize the accurate evaluation of the motion accuracy of the treatment couch in the three‐dimensional direction under single gantry angle. When performing the rotation center test of the treatment couch, our program only designs the rotation of 10 degrees for saving time, this may reduce the calculation accuracy.

Although many literatures have proved the feasibility and high accuracy of automating QA based on EPID images,^16^, ^19^, ^21^, ^22^, ^26^, ^29^ and our system has also established a series of novel algorithm to evaluate the performance of the machine, there are still some common limitations. For example, for the positioning accuracy of jaw and MLC, we only analyzed the data in specific field and do not include the whole movement range. And many analyses are only performed at 0 degrees of gantry, without considering the influence of gravity at different gantry angles. These will be considered in our next research. At the same time, we will also consider including some special MLC test patterns to evaluate the comprehensive performance of the machine in complex intensity modulation technology.

## 5 CONCLUSIONS

In this work, we designed a QA automation system to perform routine machine QA and evaluated the performance of a new linear accelerator at semi‐annual period. The measured results were all within the thresholds recommended in the AAPM TG 142 report. This system has the ability to deliver the QA plans and acquire the EPID images automatically and render the results with uncertainty on the order of 0.1 mm. The algorithm of this system also has the advantage that it does not depend on the setup error of QA phantom. And it has the ability to complete more QA items in the shortest time in the currently published literatures.

## AUTHOR CONTRIBUTIONS

This manuscript was written by WenZhao Sun, the data were collected by SiJuan Huang and Xin Yang, the data analysis were completed by ZhongHua Shi and WenZhao Sun, the table and figure were made by Can Liao, Wei Zhang and WenZhao Sun, and the research were designed by WenZhao Sun, YongBao Li and XiaoYan Huang.

## CONFLICT OF INTEREST STATEMENT

The authors have no conflict of interest to declare.

## SUPPORTING INFORMATION

The datasets are backed up on the Research Data Deposit public platform (RDD, http://www. researchdata.org.cn/, approval number: RDDB2022361804) and are available on reasonable request.
